# *Dermatophagoides pteronyssinus lytFM* encoding an NlpC/P60 endopeptidase is also present in mite‐associated bacteria that express LytFM variants

**DOI:** 10.1002/2211-5463.12263

**Published:** 2017-07-26

**Authors:** Vivian H. Tang, Geoffrey A. Stewart, Barbara J. Chang

**Affiliations:** ^1^ Marshall Centre for Infectious Diseases Research and Training School of Biomedical Sciences The University of Western Australia Crawley WA Australia

**Keywords:** D,L‐endopeptidase, *Dermatophagoides pteronyssinus*, host, LytFM, microbes, NlpC/P60

## Abstract

The bodies and faecal pellets of the house dust mite (HDM), *Dermatophagoides pteronyssinus*, are the source of many allergenic and nonallergenic proteins. One of these, the 14‐kDa bacteriolytic enzyme LytFM, originally isolated from the spent HDM growth medium, may contribute to bacteriolytic activity previously detected by zymography at 14 kDa in the culture supernatants of some bacterial species isolated from surface‐sterilised HDM. Based on previously reported findings of lateral gene transfer between microbes and their eukaryotic hosts, we investigated the presence of *lytFM* in the genomes of nine Gram‐positive bacteria from surface‐sterilised HDM, and the expression by these isolates of LytFM and its variants LytFM1/LytFM2. The *lytFM* gene was detected by PCR in the genomes of three of the isolates: *Bacillus licheniformis* strain 1, *B. licheniformis* strain 2 and *Staphylococcus aureus*. Expression of the variant LytFM1 was detected in culture supernatants of these bacteria by mass spectrometry (MS) and ELISA, and the bacterial LytFM proteins were shown by zymography to be able to hydrolyse peptidoglycan. Our previous reports of LytFM homologues in other mite species and their phylogenetic analysis had suggested that they originated from a common mite ancestor. The phylogenetic analysis reported herein and the detection of other *D. pteronyssinus* proteins by MS in the culture supernatants of the three isolates that secreted LytFM1 further support the hypothesis of lateral gene transfer to the bacterial endosymbionts from their HDM host. The complete sequence homology observed between the genes amplified from the microbes and those in their eukaryotic host indicated that the lateral gene transfer was an event that occurred recently.

**Abbreviations**
BSA: bovine serum albumin
CSL: Commonwealth Serum Laboratories
DAP: diaminopimelic acid
EIS: electrospray ionisation mass spectrometry
gDNA: genomic DNA
HDM: house dust mite
HEWL: hen egg white lysozyme
MS: mass spectrometry
TSB: tryptone soy broth

The house dust mite (HDM), *Dermatophagoides pteronyssinus*, and related species are associated with clinical allergy, and their bodies and faecal pellets are sources of many allergenic and nonallergenic proteins 1, 2, 3. These include enzymes that function on the cuticle during the moulting process, regulate the turnover of structural components during morphogenesis and growth 4, 5 and digestive enzymes produced by the salivary glands and the midgut 3, 6. One of the enzymes found in the faecal pellets is a 14‐kDa bacteriolytic enzyme designated LytFM 7, 8. Sequence data indicate that LytFM is a member of the NlpC/P60 family, which belongs to the peptidase CO clan 9. It is a single domain (P60 domain) protein with putative D,L‐endopeptidase activity that cleaves the bacterial peptidoglycan bridging peptide between the second and third residues to release the peptide L‐Ala‐D‐Glu and diaminopimelic acid or Lys 8.

As mites do not possess peptidoglycan, LytFM possibly plays a role in defence against potential pathogens, including activation of innate immunity, or in the utilisation of bacteria (endosymbionts or ingested) for food, or both 8. This is based on the observation that bacteriolytic activity, originally classified as lysozyme, detected in the spent growth medium of *D. pteronyssinus* correlated positively with the increase in number of the HDM following rearing on a diet containing *Micrococcus lysodeikticus*, a Gram‐positive bacterial species particularly susceptible to lysis. In contrast, the ‘lysozyme’ activity in whole mite extracts of the HDM was inversely correlated with that in their spent growth medium, suggesting a digestive function in the gut and a defensive function in other parts of the body such as the haemolymph and fat body 10.

Homology studies have indicated an evolutionary link between LytFM homologues of mites and bacteria 7, 8, 11, and it is possible that the mite genes originated from either endosymbionts or pathogens or from environmental species associated with food ingested by the mites. It is also possible that the LytFM enzyme is produced by mite‐associated bacteria as zymographic studies have demonstrated the presence of bacteriolytic proteins at a molecular weight corresponding to the enzyme found in both whole mite extracts 7 and bacterial culture supernatants from HDM‐associated isolates, as well as from house dust samples collected from homes of asthmatic children 12. However, although a range of bacterial species found in association with mites have been described (reviewed by Ref. 13) and some of these are known to possess genes encoding proteins containing an NlpC60/P60 domain, a single domain equivalent to the mite enzyme has not been reported.

We have examined some of the bacterial species previously isolated from mites 12 for the presence and expression of the mite gene, as it is possible that the gene encoding LytFM could have been transferred to particular strains, as the presence of a eukaryotic gene in bacterial species coexisting within their hosts has been reported previously 14, 15, 16. We have used PCR with *lytFM*‐specific primers, zymographic analyses, mass spectrometric analyses of secreted bacterial products and enzyme‐linked immunosorbent assay (ELISA) using antisera raised against LytFM sequence‐based peptides to assess this possibility. The results suggest that lateral gene transfer between HDM and its bacterial endosymbionts has recently occurred.

## Materials and methods

The HotStarTaq Master Mix Kit and QIAQuick Gel Extraction Kit were from Qiagen (Hilden, Germany), all PCR primers were from Invitrogen (Mulgrave, Vic., Australia), the *D. pteronyssinus* cDNA library was kindly provided by Wayne Thomas from the Telethon Institute for Child Health Research (Perth, Western Australia, Australia), and *D. pteronyssinus* spent growth medium (SGM) was a kind gift from the Commonwealth Serum Laboratories (Vic., Australia). Other reagents used included tryptone soy broth (TSB) (Becton Dickinson, Franklin Lakes, NJ, USA), ammonium sulfate, Bradford reagent, hen egg white lysozyme (HEWL), Triton X‐100 and TWEEN^®^ 20, which were obtained from Sigma‐Aldrich, 10 mL Bio‐Gel^®^P‐6DG gel desalting columns (BioRAD Laboratories Pty. Ltd., Gladesville, Australia), acetone (BDH, Poole, UK), biotinylated goat anti‐rabbit IgG secondary antibody and horseradish peroxidase‐conjugated streptavidin (KPL, Gaithersburg, MD, USA) and K Blue substrate (Elisa Systems, Windsor, Qld, Australia).

### Preparation of pure genomic DNA for PCR

The genomic DNA of each HDM‐associated bacterial isolate was purified as described previously 12, and its concentration was determined as described 17.

### PCR screening of the genomes of the nine HDM‐associated bacterial isolates

PCR was performed using the HotStarTaq Master Mix Kit as described previously 12 in a thermal cycler (Model PTC 200 Engine Version 4.0; MJ Research, Inc., Waltham, MA, USA) and every amplification was repeated twice. Results reported in this study were obtained with PCR using the primer set GSUTR1/GSR3 (5′‐CTATTATGAAATTCTTCTTCACT‐3′ and 5′‐TTACCAACATCGTGCAACATTAGC‐3′) 12.

### Preparation of PCR products for DNA sequencing and analysis of sequencing data

Amplicons to be sequenced were prepared using the QIAQuick Gel Extraction Kit. DNA sequencing was performed by the Lotterywest State Biomedical Facility Genomics at the Royal Perth Hospital, Perth, Australia. Analyses of sequencing data were performed as described previously 8.

### *Dermatophagoides pteronyssinus* SGM and bacterial culture supernatants

Soluble proteins were extracted from SGM as described previously 7. The HDM‐associated bacterial isolates, *Bacillus cereus*,* B. licheniformis* strain 1, *B. licheniformis* strain 2, *B. licheniformis* strain 3, *B. licheniformis* strain 4, *Micrococcus luteus*,* S. aureus*,* Staphylococcus epidermidis* and *Staphylococcus capitis* were grown at 37 °C in TSB to either the stationary or death phase of the growth curve before their culture supernatants were obtained as described previously 12. A LytFM‐enriched preparation was prepared by batch cation‐exchange chromatography and gel filtration chromatography of SGM.

### Concentration of bacterial culture supernatants by ammonium sulfate precipitation

The bacterial culture supernatants obtained at stationary phase were concentrated by adding saturated ammonium sulfate to achieve an 80% saturation. The proteins were precipitated overnight at 4 °C and recovered by centrifugation at 4 °C for 10 min at 28 000 ***g***. The resultant pellet was resuspended in 0.01 m sodium phosphate buffer, pH 6.2 to 1/10 of the volume of the original supernatant, and the sample was desalted by gel filtration chromatography on a 10‐mL Bio‐Gel^®^ P‐6DG column (BioRAD) equilibrated with 10 mm sodium phosphate, pH 7.0, containing 10 mm NaCl. Fractions collected from the column were stored at −20 °C until analysed by zymography and MS.

### Concentration of bacterial culture supernatants by acetone precipitation

The bacterial culture supernatants obtained at stationary and death phases were concentrated by adding nine volumes of acetone at −20 °C to one volume of culture supernatant and the proteins precipitated overnight at 4 °C. The proteins were subsequently recovered by centrifugation at 28 000 ***g*** for 30 min at 4 °C and the resultant pellet reconstituted in 0.01 m sodium phosphate buffer, pH 6.2 to 1/10 of the volume of the original supernatant. The samples were stored at −20 °C until analysed by ELISA and inhibition ELISA.

### Determination of protein concentrations

Protein concentrations were determined using the Bradford reagent and bovine serum albumin (BSA) as a standard.

### Zymographic analysis of bacteriolytic activity

Following desalting, fractions of the concentrated bacterial culture supernatants of the HDM‐associated isolates obtained at stationary phase were analysed by zymography as described previously 17, 18. Following SDS/PAGE, gels were rinsed in deionised water before incubation in 25 mm Tris/HCl buffer, pH 6.2 containing 1% (v/v) Triton X‐100 at 37 °C overnight. Assays were repeated with the addition of 3.2 mm DTT to 0.1 m phosphate buffer, pH 6.2, according to the method previously reported 7.

### Silver staining and trypsin digestion

Following SDS/PAGE, the gels were silver‐stained as described previously 19 before the targeted protein bands were excised and pooled for trypsin digestion by Proteomics International Pty Ltd as described 20.

### Mass spectrometry (MS)

MS was performed as described in www.proteomics.com and Bringans *et al*., 20 by Proteomics International Pty Ltd. Tryptic peptides were analysed by electrospray ionisation mass spectrometry (EIS) using the Ultimate 3000 nano HPLC system (Dionex, Sunnyvale, CA, USA) coupled to a 4000 Q TRAP mass spectrometer (Applied Biosystems, Foster City, CA, USA). In the analysis, the first run of standard EIS identified the time point at which the peptide of interest was eluted during HPLC. Subsequently, a second run of EIS focusing on a selected number of most intense peaks obtained in the first run (with peaks known to be trypsin disregarded) generated the MS/MS spectrum. The MS/MS data were submitted to mascot sequence matching software (Matrix Science, Boston, MA, USA) for comparison of the sequence of the peptide analysed with theoretical sequences found in the Ludwig NR (http://www.matrixscience.com/help/seq_db_setup_nr.html).

### Preparation of rabbit polyclonal anti‐peptide antisera against LytFM1 peptides

Anti‐peptide antisera were commercially generated using peptides synthesised by GL Biochem Ltd (Melbourne, Vic., Australia) as described previously 21. Four potential peptide candidates were identified (Fig. S4) 22, namely MKFFFTLALFCTLAISQVYC (1–20), SWGGGGIHGKSRGIGEGANT (38–57), PHTGTKVREENIGGD (127–141) and GQYSDPKCHHVAYGSHQPGD (84–103). Peptides GQYSDPKCHHVAYGSHQPGD (84–103) and PHTGTKVREENIGGD (127–141) were chosen on the basis of minimal sequence identity with known NlpC/P60 proteins based on a 2010 BLAST search. Peptide GQYSDPKCHHVAYGSHQPGD (84–103) was conjugated without modification to BSA, whereas PHTGTKVREENIGGD (127–141) was conjugated using an additional N‐terminal cysteine residue. The location of these two peptides (including the LytFM and LytFM2 variants, PHTGTNVREENIWSD (127–141) and PHTGTKVREENIWSD (127–141), respectively) was modelled using PHYRE2 23, and CHIMERA 24 was used to superimpose the appropriate pairs of structures.

### ELISA

The wells of a 96‐well‐flat‐bottom high‐binding ELISA plate (Greiner Bio‐One, Frickenhausen, Germany) were coated with 100 μL of *D. pteronyssinus* SGM or bacterial culture supernatants prepared in 0.1 m alkaline carbonate buffer, pH 9.6. Following incubation at 4 °C overnight, the wells were washed with 100 μL of wash buffer [1 × PBS containing 0.05% (v/v) TWEEN^®^ 20] and blocked with 100 μL of blocking buffer [wash buffer containing 0.05% (v/v) BSA]. The wells were washed before 100 μL of the anti‐peptide antisera diluted in blocking buffer was added. Subsequently, 100 μL of biotinylated goat anti‐rabbit IgG antiserum diluted 1/5000 in blocking buffer was applied prior to 100 μL of horseradish peroxidase‐conjugated streptavidin diluted 1/4000 in blocking buffer. Immunoreactivity was observed with the addition of 100 μL of K Blue Peroxidase substrate and the reaction terminated with 100 μL of 1.0 m HCl before the absorbance values were determined at 450 nm using a spectrophotometer (SpectraMax 190; Molecular Devices, Sunnyvale, CA, USA).

### Inhibition ELISA

Serially diluted samples of *D. pteronyssinus* SGM or bacterial culture supernatants were prepared at 10 μg·mL^−1^ in blocking buffer. One hundred microlitre of the serially diluted samples was then mixed with 100 μL of anti‐peptide antisera diluted 1/10 000 in blocking buffer and incubated for 1 h at room temperature. The samples were subsequently transferred into the wells of an ELISA plate that had been coated with 100 μL of *D. pteronyssinus* SGM diluted to 1 μg·mL^−1^ in 0.1 m alkaline carbonate buffer, pH 9.6 and blocked with blocking buffer. Goat anti‐rabbit biotinylated IgG secondary antibody was applied prior to the addition of horseradish peroxidase‐conjugated streptavidin. Following development with K Blue peroxidase substrate, the percentage inhibition was determined using the formula [1−(A_450_ of sample−A_450_ of blank)/(A_450_ of negative control−A_450_ of blank)] × 100%.

### Phylogenetic analysis

The phylogenetic relationship of the bacterial and mite LytFM proteins with the NlpC/P60 proteins of firmicutes, actinomycetes, ascomycetes and other eukaryotes used in a previous phylogenetic study 8 was analysed as described previously 8.

## Results

### The presence of *lytFM* in the genomes of the HDM‐associated *B. licheniformis* and *S. aureus* strains

**Figure 1:**
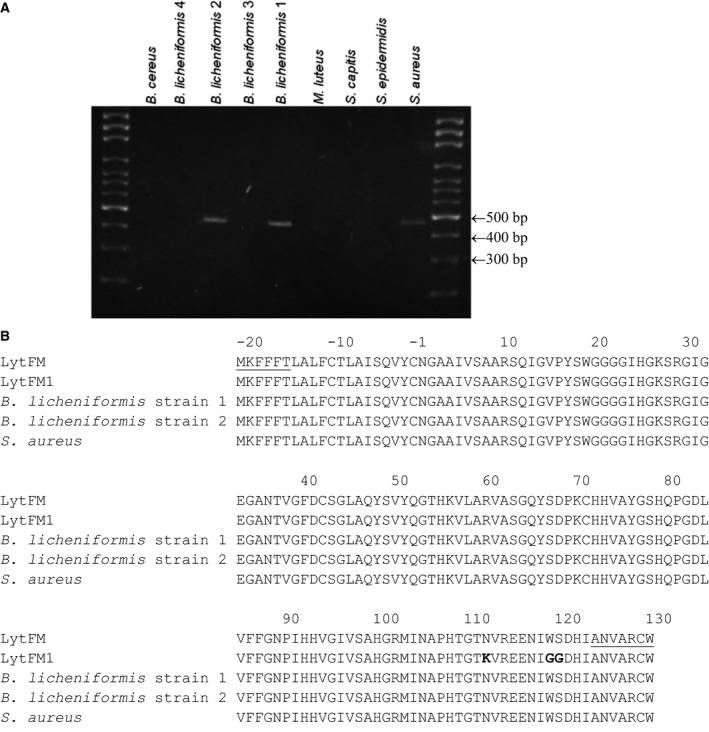
Screening of the genomes of nine HDM‐associated bacterial isolates for the presence of the *lytFM* gene. (A) PCR was performed with the primer set GSUTR1/GSR3, and an amplicon was only obtained from *Bacillus licheniformis* strain 1, *B. licheniformis* strain 2 and *Staphylococcus aureus*. (B) The amplicons were sequenced and their translated amino acid sequences aligned with those of LytFM (accession number KF113885) and LytFM1 (accession number AF409109). The amino acid residues underlined show the binding sites for the forward primer GSUTR1 (residues ‐20 to ‐15) and reverse primer GSR3 (residues 124–130). Nonhomologous residues are highlighted in boldface.

Following optimisation of PCR conditions using the primer set GSUTR1/GSR3, amplicons of the appropriate size were obtained from *B. licheniformis* strain 1 (GenBank: KU589271), *B. licheniformis* strain 2 (GenBank: KU589272) 12 and *S. aureus* (GenBank: KU589273), but not from *B. cereus*,* B. licheniformis* strain 3, *B. licheniformis* strain 4, *M. luteus*,* S. epidermidis* and *S. capitis* (Fig. 1A). The amplicons were sequenced and the deduced amino acid sequences are shown in Fig. 1B. Amplicons were not detected in the absence of template nor in the absence of primers (Fig. S1).

### Detection of 14‐kDa bacteriolytic activity in the culture supernatants of HDM‐associated *B. licheniformis* and *S. aureus*

**Figure 2:**
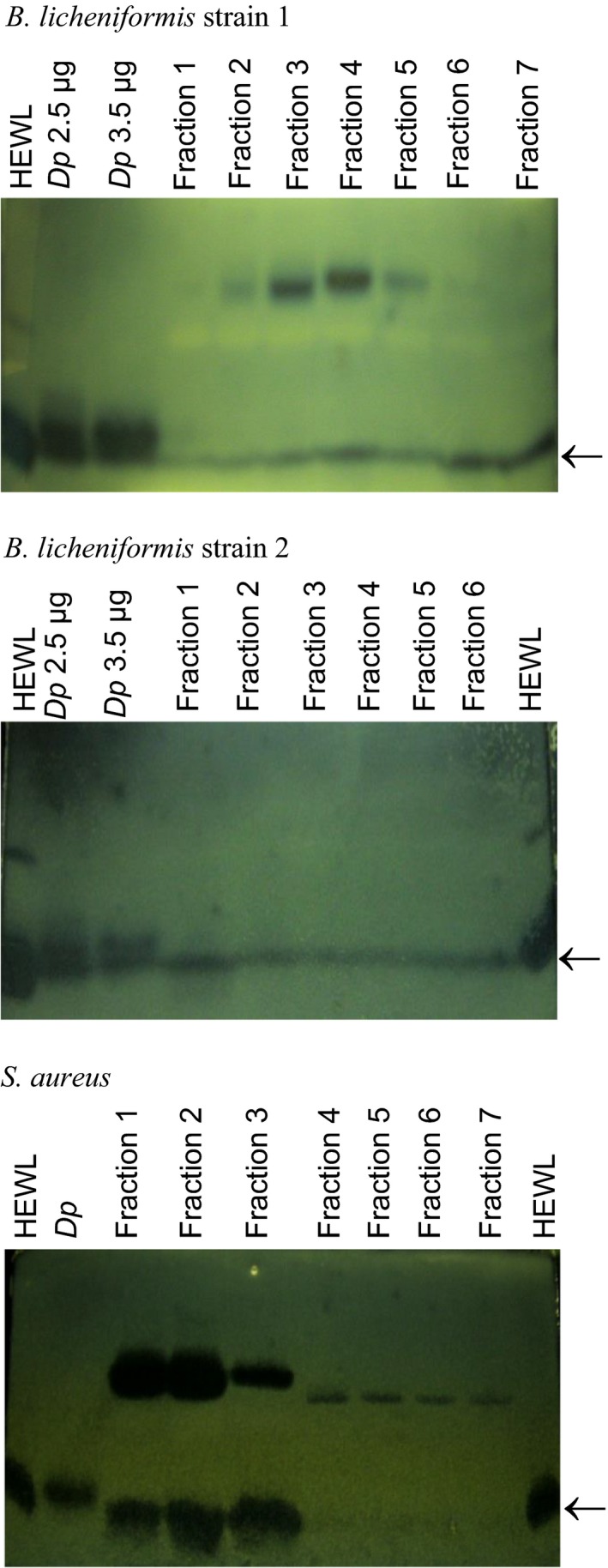
Zymographic analysis of bacteriolytic activity in bacterial culture supernatants. Following concentration by 80% saturated ammonium sulfate precipitation and desalting of the supernatants of the HDM‐associated *Bacillus licheniformis* strain 1, *B. licheniformis* strain 2 and *Staphylococcus aureus* by gel filtration chromatography, bacteriolytic activity was detected in the first six/seven fractions, including activity at 14 kDa as indicated by arrow. The bacteriolytic activity of HEWL and *Dermatophagoides pteronyssinus* SGM served as the size marker and positive control, respectively. *D. pteronyssinus* SGM (*Dp*).

When bacteriolytic activity of the nine isolates was previously investigated with zymography, very faint lytic bands were observed at 14 kDa in the culture supernatants of *B. licheniformis* strain 1 and *B. licheniformis* strain 2 12. These studies were repeated herein. The bacterial culture supernatants of the nine isolates collected at stationary phase were concentrated by 80% saturated ammonium sulfate precipitation and desalted by gel filtration chromatography before the resultant first six or seven fractions were analysed. Using *D. pteronyssinus* SGM as a positive control and HEWL as the size marker, bacteriolytic activity in the region of 14 kDa was detected in the culture supernatants of *B. licheniformis* strain 1, *B. licheniformis* strain 2 and *S. aureus* (Fig. 2), but not in the other six isolates (Fig. S2). The activity was observed in the absence of DTT, not in its presence (data not shown).

### Detection of the LytFM variants, LytFM1/LytFM2, in HDM‐associated *B. licheniformis* and *S. aureus*

**Figure 3:**
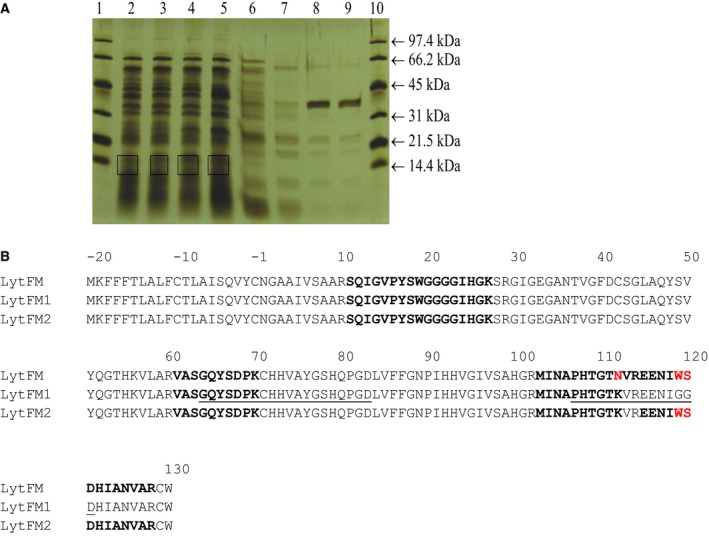
Detection of LytFM in bacterial culture supernatants of *Bacillus licheniformis* and *Staphylococcus aureus*. (A) A representative gel of *B. licheniformis* strain 2 showing the fractions obtained from the concentrated and desalted supernatant and the regions (as marked by boxes) pooled for MS analysis. (B) Alignment of the amino acid sequences of LytFM, LytFM1, LytFM2 and the peptides detected in the culture supernatants of *B*. *licheniformis* strain 1, *B. licheniformis* strain 2, *S. aureus* and *Dermatophagoides pteronyssinus* SGM which are highlighted in boldface. Nonhomologous residues are highlighted in red. The peptides used for generating anti‐peptide antisera Rb1001 and Rb999 are underlined.

Whether LytFM was expressed and secreted by the three isolates described above was ascertained by subjecting concentrated and desalted samples of bacterial culture supernatants to MS after separation by SDS/PAGE. Bands in the region of 14 kDa as shown in a representative polyacrylamide gel of concentrated culture supernatant of *B. licheniformis* strain 2 were pooled and subjected to tryptic digestion (Fig. 3A). Table 1 shows the peptides detected in supernatants of *B. licheniformis* strain 1, *B. licheniformis* strain 2 and *S. aureus*, respectively. Two peptides, namely SQIGVPYSWGGGGIHGK (11–27) and MINAPHTGTK (103–112), were detected in the culture supernatants of all three strains (Fig. S3B–D), which corresponded to amino acid sequence positions 11–27 and 103–112, respectively, in LytFM1 (Fig. 3B).

**Table 1:** LytFM and its variants detected by MS in the SGM of *Dermatophagoides pteronyssinus* and bacterial culture supernatants of *Bacillus licheniformis* strain 1, *B. licheniformis* strain 2 and *Staphylococcus aureus*

Source of peptide sequence	Peptide sequence detected	Amino acid	LytFM variants with complete sequence homology to peptides
*D. pteronyssinus* SGM	MINAPHTGTK	103–112	LytFM and LytFM2
	VASGQYSDPK	61–70	
	SQIGVPYSWGGGGIHGK	11–27	
	MINAPHTGTNVR	103–114	
	EENIWSDHIANVAR	115–128	
	MINAPHTGTNVREENIWSDHIANVAR	103–128	
*B. licheniformis* strain 1	SQIGVPYSWGGGGIHGK	11–27	LytFM1 or LytFM2
	MINAPHTGTK	103–112	
*B. licheniformis* strain 2	SQIGVPYSWGGGGIHGK	11–27	LytFM1 or LytFM2
*S. aureus*	SQIGVPYSWGGGGIHGK	11–27	LytFM1 or LytFM2
	MINAPHTGTK	103–112	

MS analysis of the corresponding SDS/PAGE bands obtained from *D. pteronyssinus* SGM revealed peptides VASGQYSDPK (61–70) and MINAPHTGTK (103–112) (Table 1, Fig. S3A). A subsequent MS study using a LytFM‐enriched SGM‐derived sample revealed several peptides including VASGQYSDPK (61–70) and MINAPHTGTK (103–112) as described above, as well as SQIGVPYSWGGGGIHGK (11–27), MINAPHTGTNVR (103–114), EENIWSDHIANVAR (115–128) and MINAPHTGTNVREENIWSDHIANVAR (103–128) (Table 1). The sequences from the bacterial samples corresponded to either the LytFM1 or LytFM2 variants, whereas the mite‐derived sequences corresponded to the LytFM and LytFM2 variants (Fig. 3B).

A summary of proteins other than LytFM1/2 detected in the culture supernatants is shown in Table S1: these included allergens and a protease of *D. pteronyssinus*, as well as proteins from a variety of bacterial species.

### LytFM of HDM‐associated *B. licheniformis* strain 1, *B. licheniformis* strain 2 and *S. aureus* verified by anti‐peptide antisera Rb1001 and Rb999

**Figure 4:**
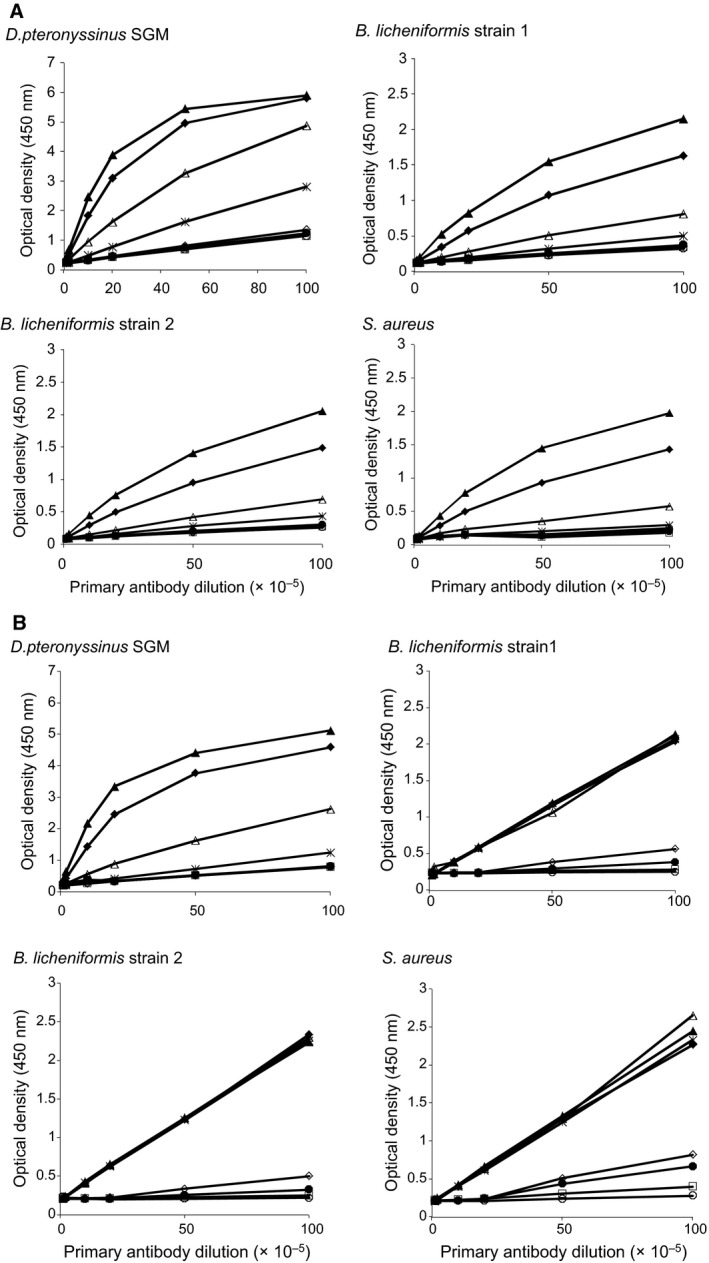
Immunoreactivity of anti‐peptide antisera Rb1001 (A) and Rb999 (B) with *Dermatophagoides pteronyssinus* SGM and bacterial culture supernatants of the HDM‐associated *Bacillus licheniformis* strain 1, *B. licheniformis* strain 2 and *Staphylococcus aureus*. Both the polyclonal anti‐peptide antisera reacted with 0.01 μg (*), 0.1 μg (▵), 1 μg (♦) and 5 μg (▲) of protein found in the *D. pteronyssinus* SGM or bacterial culture supernatants. Preimmune sera Rb 1001 and Rb999 were used as the respective negative controls and were tested using 0.01 μg (○), 0.1 μg (□), 1 μg (●) and 5 μg (♢) of protein in the *D. pteronyssinus* SGM or bacterial culture supernatants. Assays were performed in duplicate, and mean optical density values are shown.

**Figure 5:**
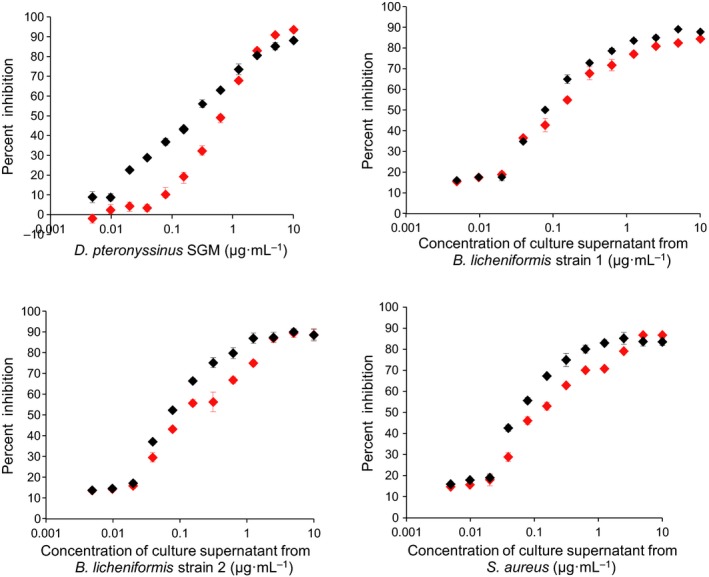
Inhibition of anti‐peptide antisera Rb1001 (in red) and Rb999 (in black) by *Dermatophagoides pteronyssinus* SGM and the bacterial culture supernatants of the HDM‐associated *Bacillus licheniformis* strain 1, *B. licheniformis* strain 2 and *Staphylococcus aureus*. Assay for the positive control *D. pteronyssinus* SGM was performed in triplicate, and all the other assays were performed in duplicate. Mean percentage inhibition values are shown. The correlation coefficients (Rb1001/Rb999) for *D. pteronyssinus* SGM,* B. licheniformis* strain 2 and *S. aureus* are 0.93/0.99, 0.95/0.92, 0.97/0.91 and 0.97/0.90, respectively. The IC_50_, that is concentrations of *D. pteronyssinus* SGM, bacterial culture supernatants of *B. licheniformis* strain 1, *B. licheniformis* strain 2 and *S. aureus* required to generate 50% inhibition of the immunoreactivity of the anti‐peptide antisera (Rb1001/Rb999) are 0.51/0.23, 0.15/0.11, 0.17/0.1 and 0.17/0.1 μg·mL^−1^, respectively.

Polyclonal antibodies raised against peptides derived from LytFM1 (Fig. 3B, Fig. S4) were used in ELISA to determine whether LytFM could be detected in culture supernatants from any of the three bacterial isolates. Figure S5 shows the likely locations of the two peptides on the surface of a model of LytFM generated using PHYRE2 23. The top ranked model (125 residues modelled, 100% confidence) was based on the rv1477 protein of *Mycobacterium tuberculosis* 25. Anti‐peptide Rb1001 (GQYSDPKCHHVAYGSHQPGD (84–103)) and Rb999 (PHTGTKVREENIGGD (127–141)) antisera did not react with stationary‐phase culture supernatants of all nine isolates (data not shown). However, LytFM1 was detected in the *D. pteronyssinus* SGM and the culture supernatants of *B. licheniformis* strain 1, *B. licheniformis* strain 2 and *S. aureus* collected from the death phase of the growth cycle (Fig. 4A). Similar results were obtained with anti‐peptide antiserum Rb999 (Fig. 4B). Neither of the antisera reacted with death‐phase culture supernatants of any of the other six HDM‐associated isolates, namely *B. licheniformis* strain 4, *S. epidermidis*,* M. luteus*,* B. cereus*,* B*. *licheniformis* strain 3 and *S. capitis* (Fig. S6A,B). Inhibition ELISA studies showed that concentrated culture supernatants of the HDM‐associated *B. licheniformis* strain 1, *B*. *licheniformis* strain 2 and *S. aureus* inhibited both antisera with 50% inhibition achieved using extracts in the region of 0.10–0.51 μg·mL^−1^ (Fig. 5).

### Acquisition of *lytFM* and its variants by the three HDM‐associated bacterial isolates

**Figure 6:**
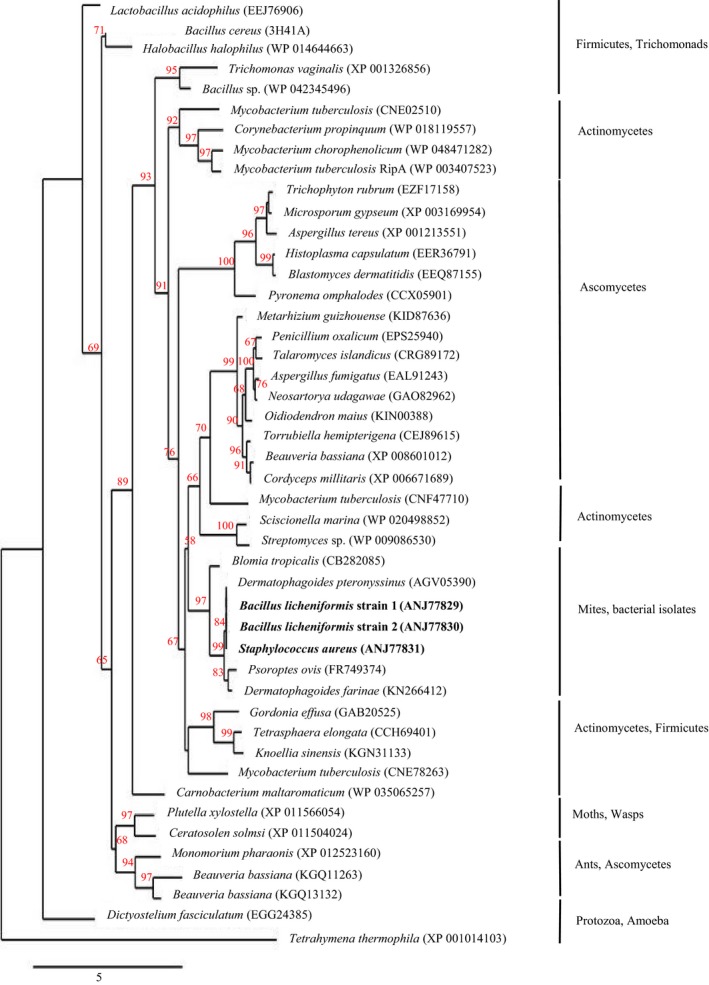
Phylogenetic relationship of mite and bacterial LytFM homologues and prokaryotic and eukaryotic NlpC/P60 proteins. According to the GenBank records, the accession numbers of *Aspergillus fumigatus* (XP 753281), *Neosartorya udagawae* (GAO84874), *Tetrahymena thermophila* (XM 001014103) and *Dictyostelium fasciculatum* (GL883007) are updated to EAL91243, GAO82962, XP 001014103 and EGG24385, respectively. Phylogenetic analysis was performed with Phylogeny.fr pipeline 26, approximate likelihood ratio test was used, and the confidence values are shown on the branches.

All the amino acid sequences of LytFM homologues included in a previous phylogenetic analysis 8 except that of *Ceratitis capitate* (XP 012159888) which has been withdrawn from GenBank were used. Phylogenetic analysis revealed clustering of LytFM homologues into distinct monophyletic groups similar to that observed in the previous analysis 8. The mite homologues and LytFM obtained from *B. licheniformis* strain 1, *B. licheniformis* strain 2 and *S. aureus* formed a clade distinct from those of other microbes, fungi and other eukaryotes (Fig. 6), indicating a possible lateral gene transfer of *lytFM* and its variants from *D. pteronyssinus* to its endosymbiotic microbes. In addition, consistent with the previous analysis 8, the mite LytFM homologues were most closely related to those of some of the actinomycetes and shared a common ancestor with both the actinomycetes and ascomycetes.

## Discussion

To investigate the possibility that the *lytFM* gene in *D. pteronyssinus* was horizontally transferred to microorganisms cohabiting within the HDM, nine Gram‐positive HDM‐associated bacteria isolated from surface‐sterilised *D. pteronyssinus* 12 were screened by PCR for the existence of *lytFM* or its variants in their genomes. The *lytFM* gene was amplified from genomic DNA of *B. licheniformis* strain 1, *B*. *licheniformis* strain 2 and *S. aureus*, but not from the other two *B. licheniformis* strains, or from the other bacterial isolates from *D. peteronyssinus* 12. Similarly, the LytFM protein was found to be present in the culture supernatants from only these three bacterial species as judged by MS (stationary‐phase samples) and by ELISA (death‐phase samples). With regard to the PCR studies, only the originally described LytFM variant 7 was distinguished in the bacterial extracts, in contrast to the detection of this and the other two variants distinguished in other molecular studies 8. However, MS detected peptides from all the three possible variants thus far described.

The results obtained in this study verified the contribution of LytFM or its variants, LytFM1/LytFM2, to bacteriolytic activity in the *D. pteronyssinus* SGM as indicated previously by zymographic analysis at 14 kDa 7. Faint lytic bands at 14 kDa were also previously reported in a zymographic analysis of culture supernatants of HDM‐associated isolates *B. licheniformis* strain 1 and *B*. *licheniformis* strain 2 12. Concentration of the bacterial culture supernatants by 80% saturated ammonium sulfate precipitation resulted in enhanced bacteriolytic activity observed at about 14 kDa in the culture supernatants of *B. licheniformis* strain 1, *B*. *licheniformis* strain 2 and *S. aureus* in the present study in the absence of DTT but not its presence. These data are in contrast to those from previous findings that bacteriolytic activity in *D. pteronyssinus* SGM is enhanced by DTT as determined using a plate lytic assay 7 and that bacteriolytic activity of bacterial proteins from the NlpC/P60 superfamily is enhanced by thiols 7, 27, 28, 29.

The differences between the two systems, that is plate lytic assay applied previously 7 and zymography, possibly accounted for the phenomenon that bacteriolytic activity at 14 kDa was not observed in the samples analysed if DTT was added to the zymographic buffer. Of the three cysteine residues in LytFM, that is C42, C71 and C129 7, C42 is a highly conserved cysteine residue found in the C‐terminal region of all members of the NlpC/P60 superfamily 30 and is therefore likely to be involved in the catalytic function of the bacteriolytic enzyme. In that regard, C71 likely forms a disulfide bond with C129 7. In a zymographic analysis, following SDS/PAGE during which proteins were separated in a denatured form, immersing the polyacrylamide gel in a zymographic buffer containing DTT might lead to protein renaturation that favoured the formation of disulfide bonds between C42 and C71 or C42 and C129, or both, thus inhibiting the bacteriolytic activity of LytFM.

The presence of LytFM was detected by ELISA using anti‐peptide antisera against two epitopes, namely GQYSDPKCHHVAYGSHQPGD (84–103) and PHTGTKVREENIGGD (127–141), originally designed based on the LytFM1 sequence 7. Whilst the anti‐GQYSDPKCHHVAYGSHQPGD (84–103) antiserum should recognise all three of the LytFM variants, it is possible that the observed sequence differences in the C‐terminal portions may have influenced immunoreactivity using the anti‐PHTGTKVREENIGGD (127–141) antiserum, although unlikely given recent information about epitopes and antisera 31. For example, the corresponding sequences of LytFM and LytFM2 are PHTGTNVREENIWSD (127–141) and PHTGTKVREENIWSD (127–141), respectively, highlighting variation in three residues (K versus N; and GG versus WS). Molecular superimposition modelling shows slight structural deviations with the ‘WS’ sequence compared with the ‘GG’ sequence (Fig. S5), but the N versus K substitution did not appear to markedly influence local structures. However, modelling indicates that the antisera recognise surface‐located regions as well as possessing disordered secondary structure (Fig. S5), highly predictive indicators of B‐cell epitopes 31.

The obvious question arising from the ELISA studies is whether the antisera cross‐reacted with non‐LytFM‐related bacterial proteins belonging to the NlpC/P60 family, as they are ubiquitous and are known to be present in most bacterial species including *B. licheniformis* and *S. aureus* 9, 11. As the sequences chosen for the antisera production were based on a 2010 BLAST search when limited sequence homology was detected, further sequence identity searches were undertaken (G. A. Stewart, unpublished observations) using nonspecies‐restricted pSI‐BLAST. In this regard, the peptide sequence GQYSDPKCHHVAYGSHQPGD (84–103) matched a RipB‐like protein (*E* = 0.002, 13/19 identities) from the American snowberry fruit fly (*Rhagoletis zephyria*). The complete sequences for *R. zephyria* LytFM appear to be the first reported for an insect species and are single domain proteins highly homologous to the mite sequences including those from *Dermatophagoides farinae*,* Psoroptes ovis* and *Blomia tropicalis*. From a phylogenetic perspective, the *R. zephyria* sequences are more similar to those of *B. tropicalis* than the other mite species (Fig. S7).

A search using the peptide sequence PHTGTKVREENIGGD (127–141) revealed homology with LytFM1 (*E* = 2e‐06) and LytFM (*E* = 0.035) and with a single domain hypothetical NlpC/P60 protein from the ascomycete *Torrubiella hemipterigena* (*E* = 6.3, 9/15 identities, HTGTKVREQSI (132–142)). A species‐specific search against the peptides did not reveal any perfect matches but short (4–5 contiguous amino acids) sequence stretches of homology were identified in various bacterial proteins, although none were related to members of the NlpC/P60 family. Whether such short homologous regions present in bacterial proteins confer immunoreactivity sufficient to account for the data reported in the studies here is unclear, but unlikely. For example, antibody paratopes of about 17 amino acid residues usually interact with sequences (linear or discontinuous) of about 15–22 residues within a B‐cell epitope and the 2–5 residues required for optimal binding within this sequence will be distributed throughout the epitope rather than being contiguous 31. In addition, the majority of antibodies tested against B‐cell epitopes react very poorly with peptides of between 7–12 residues and less 31.

LytFM immunoreactivity was not detected with any bacterial sample by ELISA using culture supernatants grown to stationary phase in contrast to data obtained using MS, suggesting differences in sensitivity between the two techniques. LytFM immunoreactivity was, however, readily detected in culture supernatants obtained at the death phase, suggesting that expression of this gene at this time might offer some selective advantage to the organism. For example, peptidoglycan cleavage of the cell wall of dead or dying bacteria may help viable organisms survive when nutrients are fast depleting and waste and toxin levels are rapidly increasing. Expression at an earlier time point, such as log or stationary phase, would likely interfere with peptidoglycan biosynthesis and thus cell wall integrity. In this regard, bacteriolysis has been observed in both Gram‐positive and Gram‐negative bacteria under conditions of nutrient depletion 32, 33. In addition, starved *Bacillus subtilis* has been reported to secrete lytic enzymes during the early stages of sporulation prompting the suggestion that such enzymes enable a viable population to obtain nutrients from the lysed cells to delay or avoid sporulation 34, 35.

In this study, genes *lytFM* and *lytFM1* were found associated with genomic DNA derived from two *B. licheniformis* strains and *S. aureus* isolated from surface‐sterilised laboratory‐reared *D*. *pteronyssinus*. However, whole‐genome sequencing of *B. licheniformis* and *S. aureus* strains reported to date in public databases such as GenBank has so far failed to reveal any gene encoding the mite enzyme, suggesting that the *lytFM* sequences were not amplified from the bacterial chromosome. However, they may have been amplified from an additional replicon within the mite‐associated isolates, such as a plasmid, but this remains to be determined. This is not without precedent for a protein belonging to the NlpC/P60 family as a multidomain hypothetical protein possessing a C‐terminal NlpC/P60 domain with significant homology to LytFM and its variants (*E* = 1e‐26) has been found in one of two plasmids [pKRAD01 (accession number CP000751.1)] of the Gram‐positive bacterial species *Kineococcus radiotolerans*.

The possibility of a lateral gene transfer of a mite gene and its variants to microbial endosymbionts is consistent with our previous data 8 and the phylogenetic data presented in this study showing that mite LytFM homologues form a monophyletic group with an evolutionary link to one or more prokaryotic endosymbionts. Similar phenomena involving an arthropod 16 and humans 14, 15 and their microbial endosymbionts have been reported. Transposons, direct or indirect repeats were not detected in the 5′ and 3′ flanking sequence of *lytFM* in a *D. pteronyssinus* genomic DNA library 8, suggesting no involvement of mobile genetic elements such as transposons 14, 15, 16 in the cross‐kingdom gene transfer. However, lateral gene transfer between two microbes within the HDM might also be facilitated by plasmid transfer 36, which has been reported to occur between *Bacillus* strains 37 and between *Bacillus* species and *S. aureus* 37, 38. In either scenario, the gene acquired in a plasmid by a microbe could move by recombination events into its chromosome or be further transferred to another microbe 36.

Regardless of the exact paths through which *B. licheniformis* strain 1, *B. licheniformis* strain 2 and *S. aureus* acquired the genes, the presence of a gene in a microbe that showed complete homology with that of its eukaryotic source indicates that lateral gene transfer occurred recently 14. The absence of bacterial homologues of LytFM/LytFM1/LytFM2 in any protein databases, which is in line with the findings reported previously 15, is further indication that the lateral gene transfer occurred recently. In addition, *lytFM* and its variants were not found in *B. cereus*, two other *B. licheniformis* strains and two other Staphylococcal species isolates included in this study, with similar phenomena reported previously 14, 36. In concert with data obtained from MS analysis revealing that several other *D. pteronyssinus* proteins were expressed by *B. licheniformis* strain 1, *B. licheniformis* strain 2 and *S. aureus* (Table S1), these results indicated for the first time, the possible acquisition of genes by mite‐associated bacterial species from a HDM through lateral gene transfer.

## Author contributions

VHT designed, planned and performed the experiments, acquired and analysed the data and wrote the manuscript. GAS provided the experimental reagents and materials, advised in the design and planning of experiments, contributed to data analysis and edited the manuscript. BJC contributed to the design and planning of experiments, oversaw the progress of the experimental work, contributed to data analysis and edited the manuscript.

## Supporting information


**Fig. S1.** Amplification of *lytFM* from the HDM‐associated *B*. *licheniformis* 1, *B. licheniformis* 2 (upper panel) and *S. aureus* (lower panel) using the primer set GSUTR1/GSR3 was validated by the absence of any amplicon when the PCR was repeated without any primers (‐) or when a negative control PCR was performed with the primers in the absence of any template.
**Fig. S2.** Zymographic analysis of bacteriolytic activity in bacterial culture supernatants of *B. licheniformis* strain 4, *S. epidermidis*,* M. luteus*,* B. cereus*,* B*. *licheniformis* strain 3 and *S. capitis*.
**Fig. S3.** MS detection of the two peptides M_103_INAPHTGTK_112_ and V_61_ASGQYSDPK_70_ in the *D. pteronyssinus* SGM (A), S_11_QIGVPYSWGGGGIHGK_27_ and M_103_INAPHTGTK_112_ in the culture supernatant of *B. licheniformis* 1 (B), S_11_QIGVPYSWGGGGIHGK_27_ in the culture supernatant of *B*. *licheniformis* 2 (C) and S_11_QIGVPYSWGGGGIHGK_27_ and M_103_INAPHTGTK_112_ in the culture supernatant of *S. aureus* (D).
**Fig. S4.** The antigenic indices for all the potential epitopes of LytFM1 were calculated as described previously [22] using the programs PeptideStructure and PlotStructure [21] (upper panel).
**Fig. S5.** Relative location of the two regions of LytFM used in the design of the anti‐LytFM1 peptide antisera described in this study.
**Fig. S6.** Immunoreactivity of anti‐peptide antisera Rb1001 (A) and Rb999 (B) with the bacterial culture supernatants of the HDM‐associated *B. licheniformis* strain 4, *S. epidermidis*,* M. luteus*,* B. cereus*,* B*. *licheniformis* strain 3 and *S. capitis*.
**Fig. S7.** Alignment of the amino acid sequences of *Rhagoletis zephyria* peptidoglycan endopeptidase RipB‐like protein, *D. pteronyssinus* LytFM and LytFM1 and the LytFM homologues of *B. tropicalis*,* D. farinae* and *P. ovis*.
**Table S1.** Summary of proteins other than LytFM1 present in the bacterial culture supernatants of the HDM‐associated *B. licheniformis* strain 1, *B. licheniformis* strain 2 and *S. aureus* following analysis by MS.
