# Supplementary material for: Dermatophagoides pteronyssinus lytFM encoding an NlpC/P60 endopeptidase is also present in mite‐associated bacteria that express LytFM variants
Source: FEBS Open Bio. 2017 Jul 26;7(9):1267–80. doi: 10.1002/2211-5463.12263 (PMC5586350; doi:10.1002/2211-5463.12263)
Supplement: Supplementary file 1 — Fig. S1. Amplification of lytFM from the HDM‐associated B. licheniformis 1, B. licheniformis 2 (upper panel) and S. aureus (lower panel) using the primer set GSUTR1/GSR3 was validated by the absence of any amplicon when the PCR was repeated without any primers (‐) or when a negative control PCR was performed with the primers in the absence of any template. Fig. S2. Zymographic analysis of bacteriolytic activity in bacterial culture supernatants of B. licheniformis strain 4, S. epidermidis, M. luteus, B. cereus, B. licheniformis strain 3 and S. capitis. Fig. S3. MS detection of the two peptides M103INAPHTGTK112 and V61ASGQYSDPK70 in the D. pteronyssinus SGM (A), S11QIGVPYSWGGGGIHGK27 and M103INAPHTGTK112 in the culture supernatant of B. licheniformis 1 (B), S11QIGVPYSWGGGGIHGK27 in the culture supernatant of B. licheniformis 2 (C) and S11QIGVPYSWGGGGIHGK27 and M103INAPHTGTK112 in the culture supernatant of S. aureus (D). Fig. S4. The antigenic indices for all the potential epitopes of LytFM1 were calculated as described previously [22] using the programs PeptideStructure and PlotStructure [21] (upper panel). Fig. S5. Relative location of the two regions of LytFM used in the design of the anti‐LytFM1 peptide antisera described in this study. Fig. S6. Immunoreactivity of anti‐peptide antisera Rb1001 (A) and Rb999 (B) with the bacterial culture supernatants of the HDM‐associated B. licheniformis strain 4, S. epidermidis, M. luteus, B. cereus, B. licheniformis strain 3 and S. capitis. Fig. S7. Alignment of the amino acid sequences of Rhagoletis zephyria peptidoglycan endopeptidase RipB‐like protein, D. pteronyssinus LytFM and LytFM1 and the LytFM homologues of B. tropicalis, D. farinae and P. ovis. Table S1. Summary of proteins other than LytFM1 present in the bacterial culture supernatants of the HDM‐associated B. licheniformis strain 1, B. licheniformis strain 2 and S. aureus following analysis by MS. [file FEB4-7-1267-s001.pdf]

Fig. S1

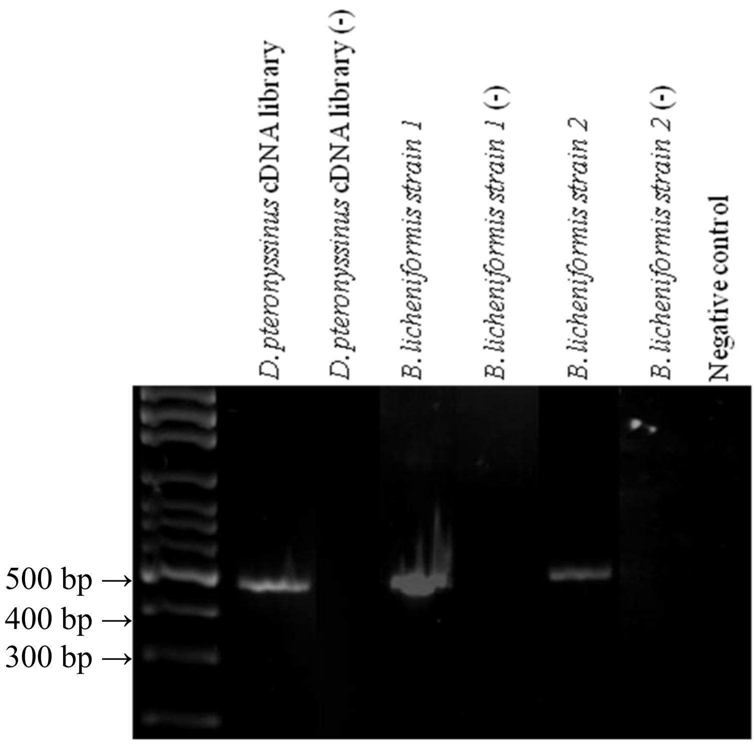
D. pteronyssinus cDNA library
D. pteronyssinus cDNA library (-)
B. licheniformis strain 1
B. licheniformis strain 1 (-)
B. licheniformis strain 2
B. licheniformis strain 2 (-)
Negative control
500 bp →
400 bp →
300 bp →

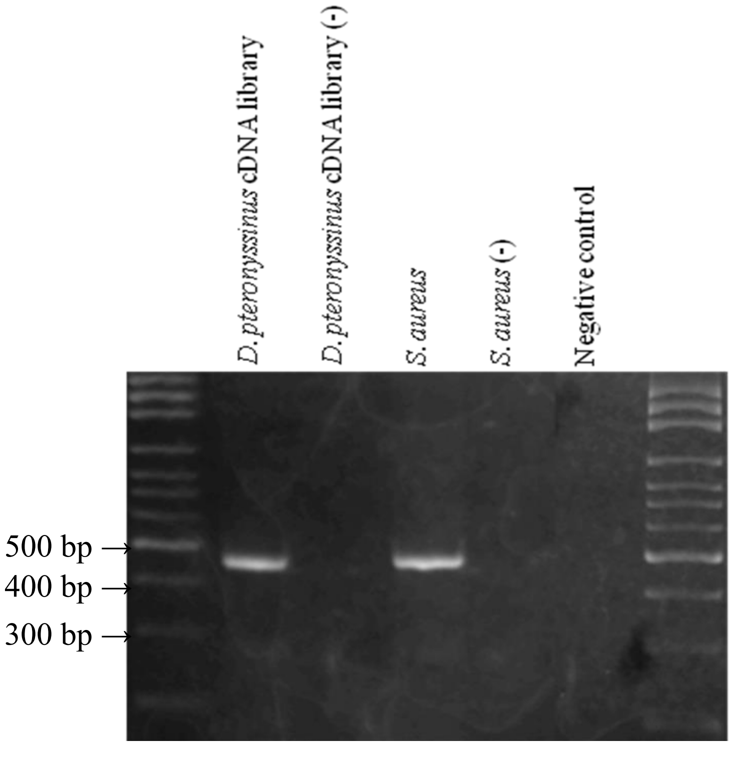
D. pteronyssinus cDNA library
D. pteronyssinus cDNA library (-)
S. aureus
S. aureus (-)
Negative control
500 bp →
400 bp →
300 bp →

Fig. S2

B. licheniformis strain 4

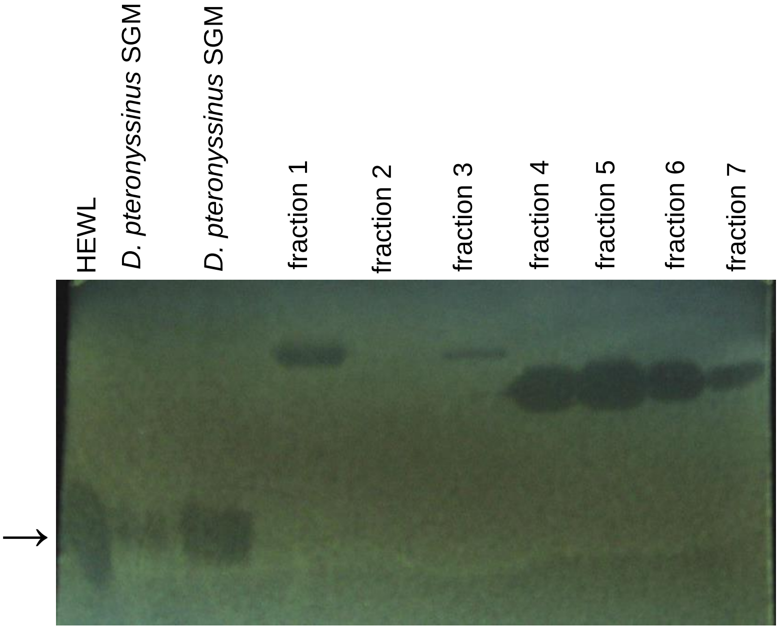

S. epidermidis

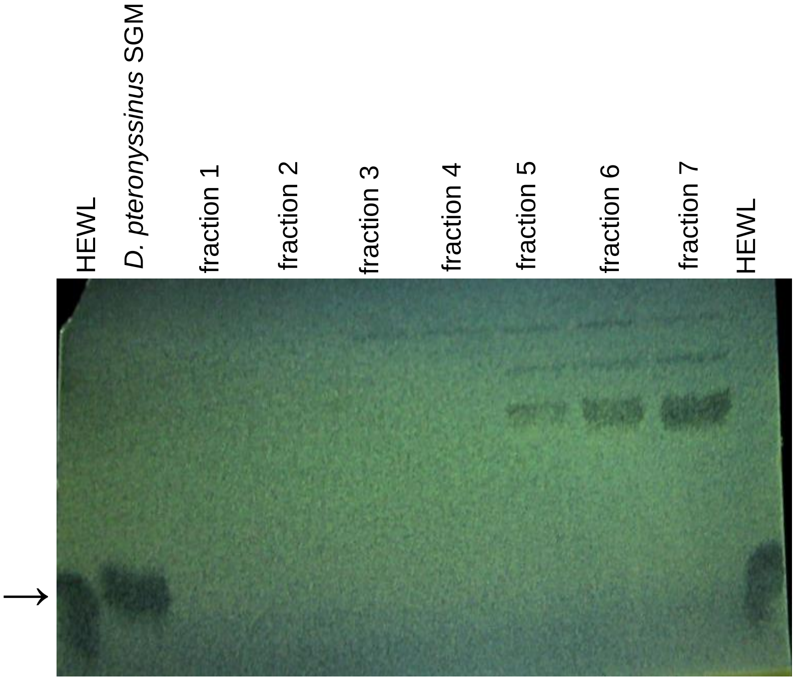

M. luteus

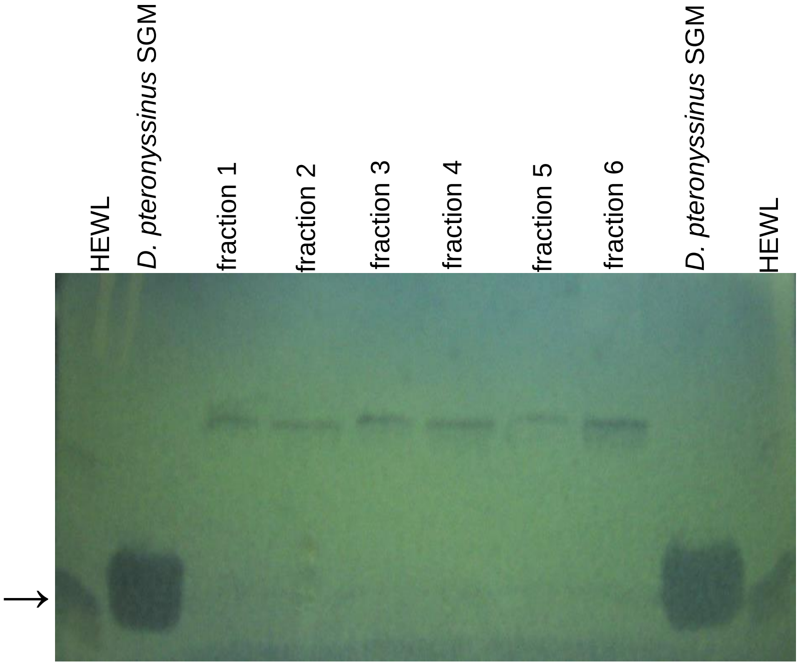

B. cereus

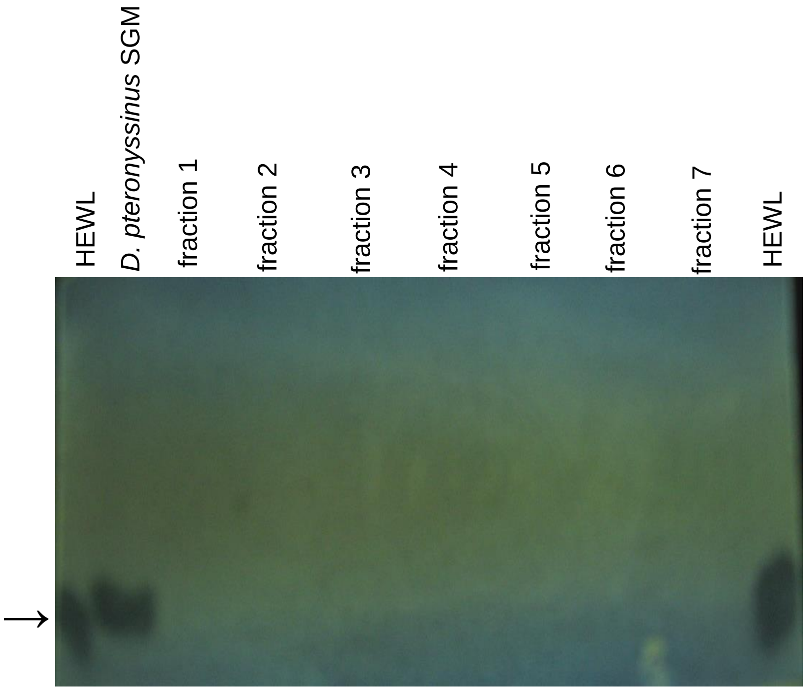

B. licheniformis strain 3

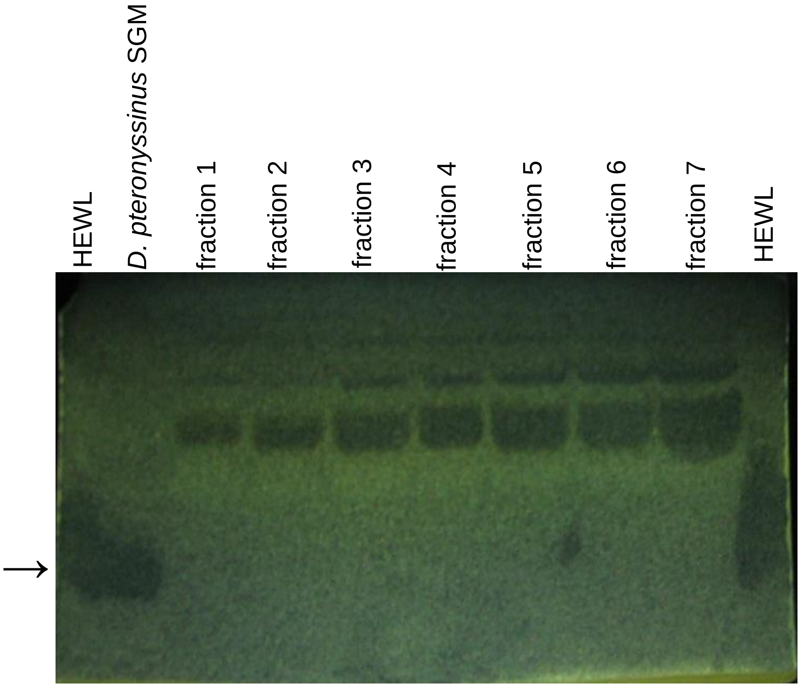

S. capitis

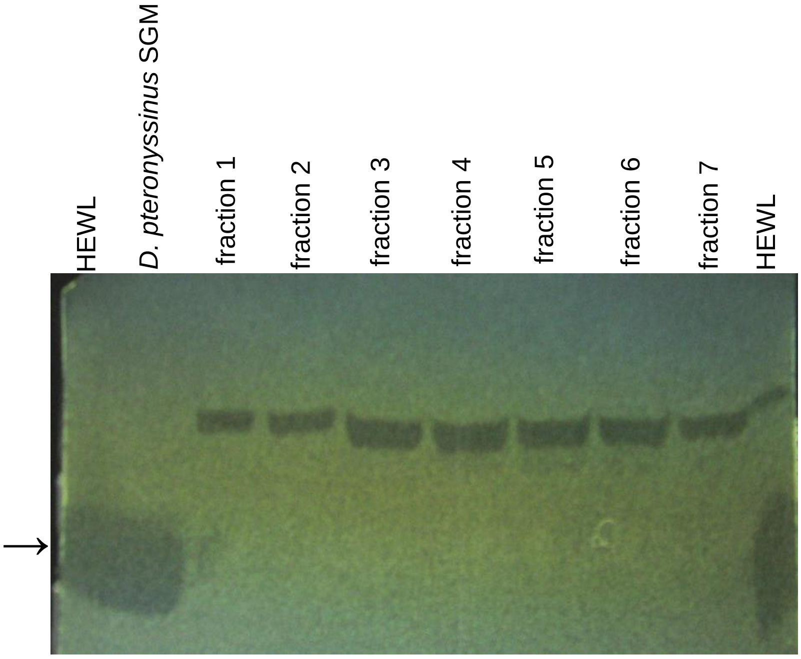

Fig. S3

A

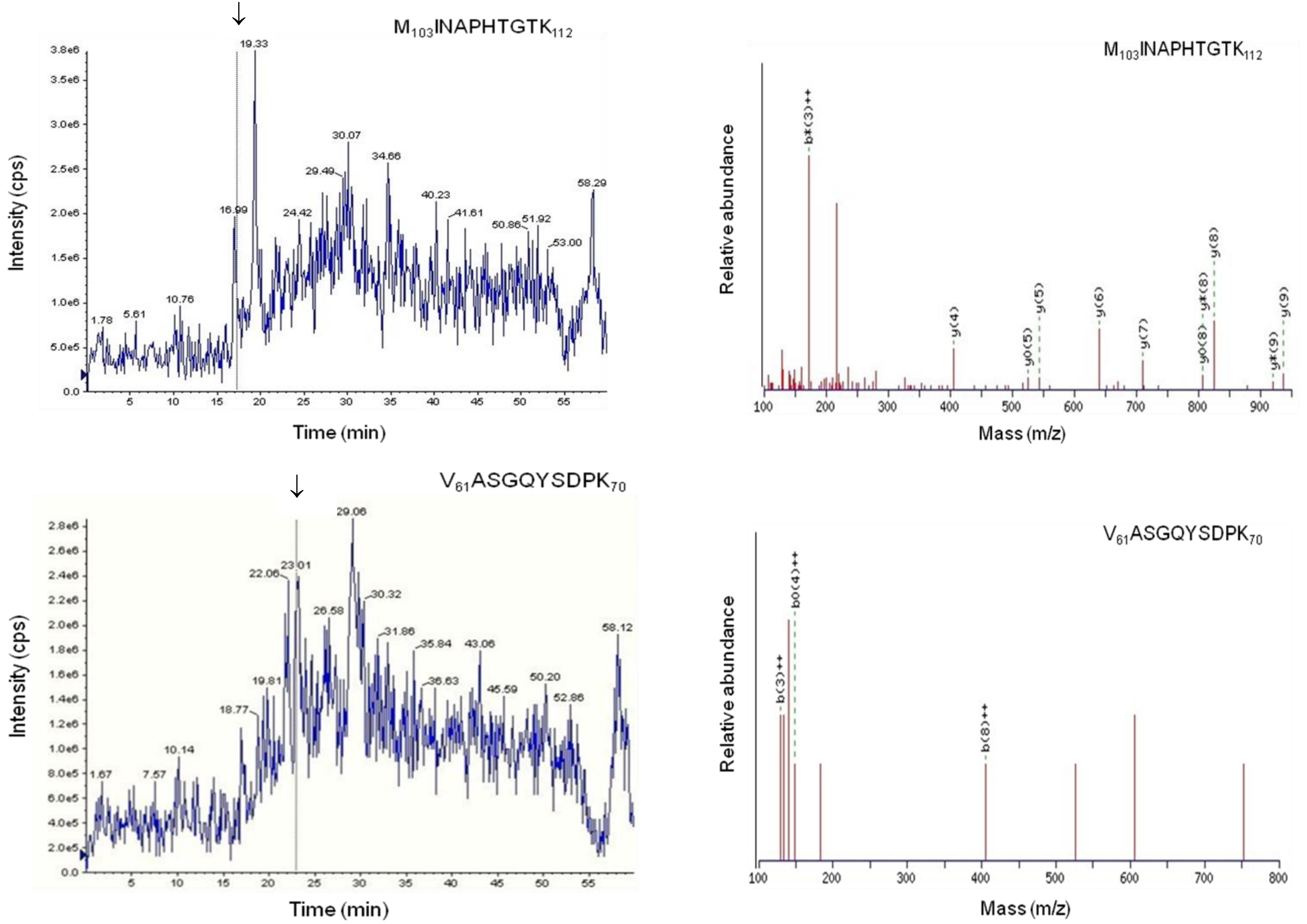

M103INAPHTGTK112
Intensity (cps)
Time (min)
19.33
16.99
24.42
29.49
30.07
34.66
40.23
41.61
50.86
51.92
53.00
58.29
1.78
5.61
10.76
Relative abundance
Mass (m/z)
b*(3)++
y(4)
y0(5)
y(5)
y(6)
y(7)
y0(8)
y*(8)
y(8)
y*(9)
y(9)
V61ASGQYSDPK70
29.06
22.06
23.01
30.32
26.58
31.86
35.84
43.06
36.63
45.59
50.20
52.86
58.12
19.81
18.77
10.14
1.67
7.57
b(3)++
b0(4)++
b(8)++

B

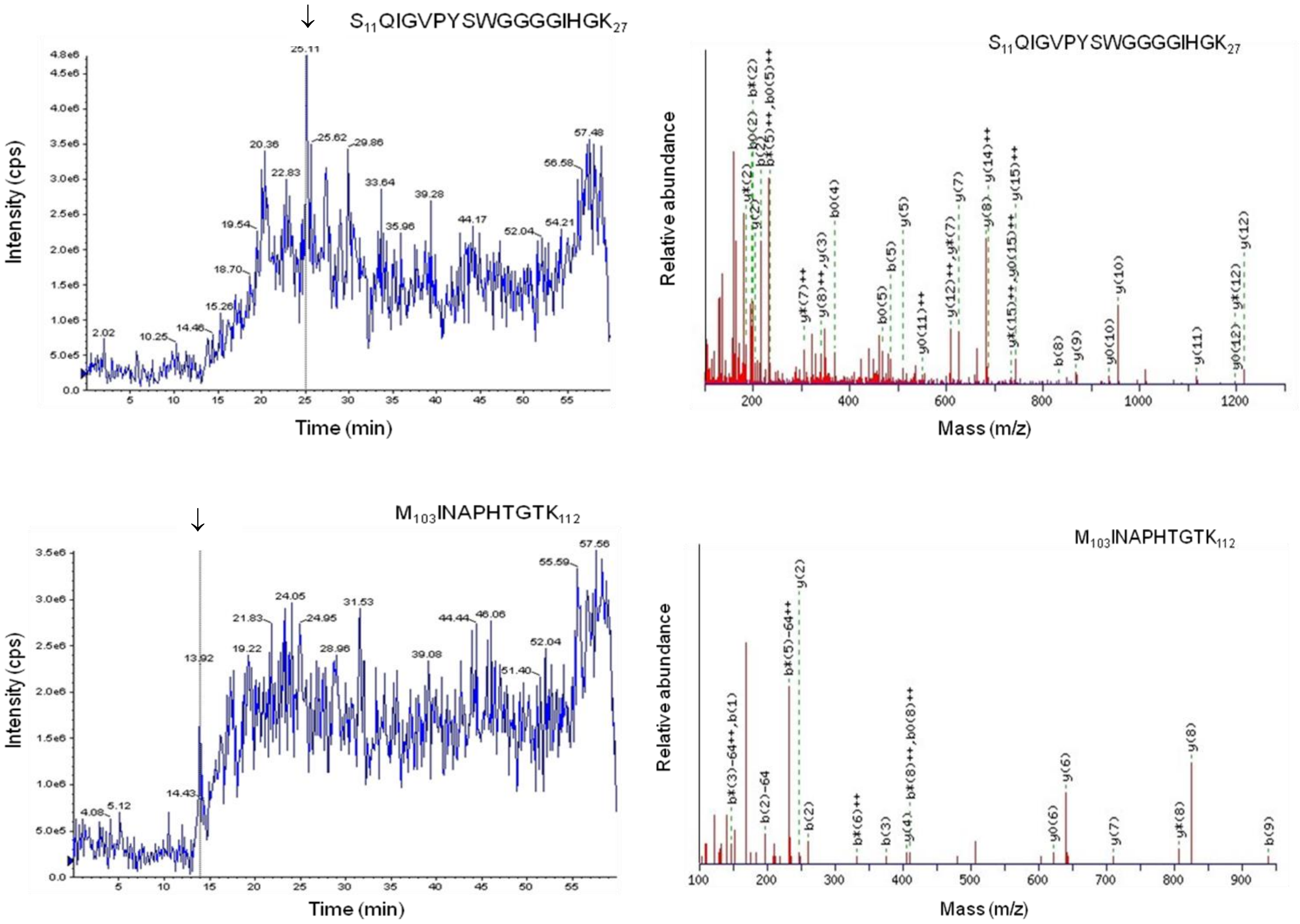
S11QIGVPYSWGGGGIHGK27
Intensity (cps)
Time (min)
Relative abundance
Mass (m/z)
M103INAPHTGTK112
Intensity (cps)
Time (min)
Relative abundance
Mass (m/z)

C

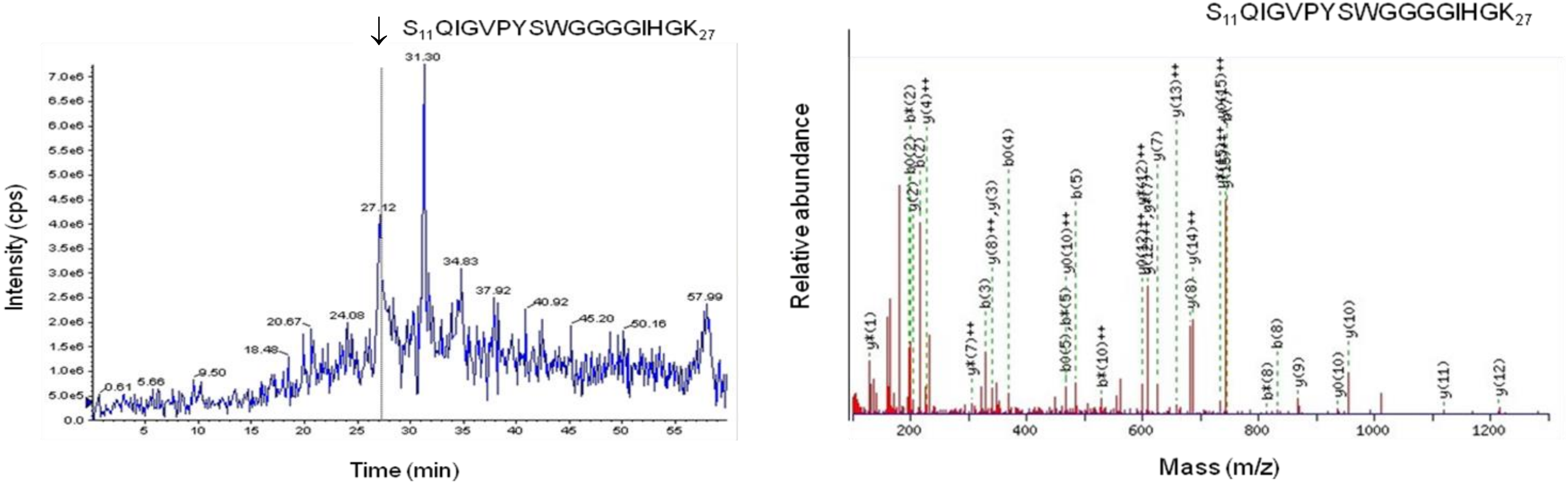

S11QIGVPYSWGGGGIHGK27
Intensity (cps)
Time (min)
31.30
27.12
34.83
37.92
40.92
45.20
50.16
57.99
24.08
20.67
18.48
9.50
5.66
0.61
S11QIGVPYSWGGGGIHGK27
Relative abundance
Mass (m/z)

D

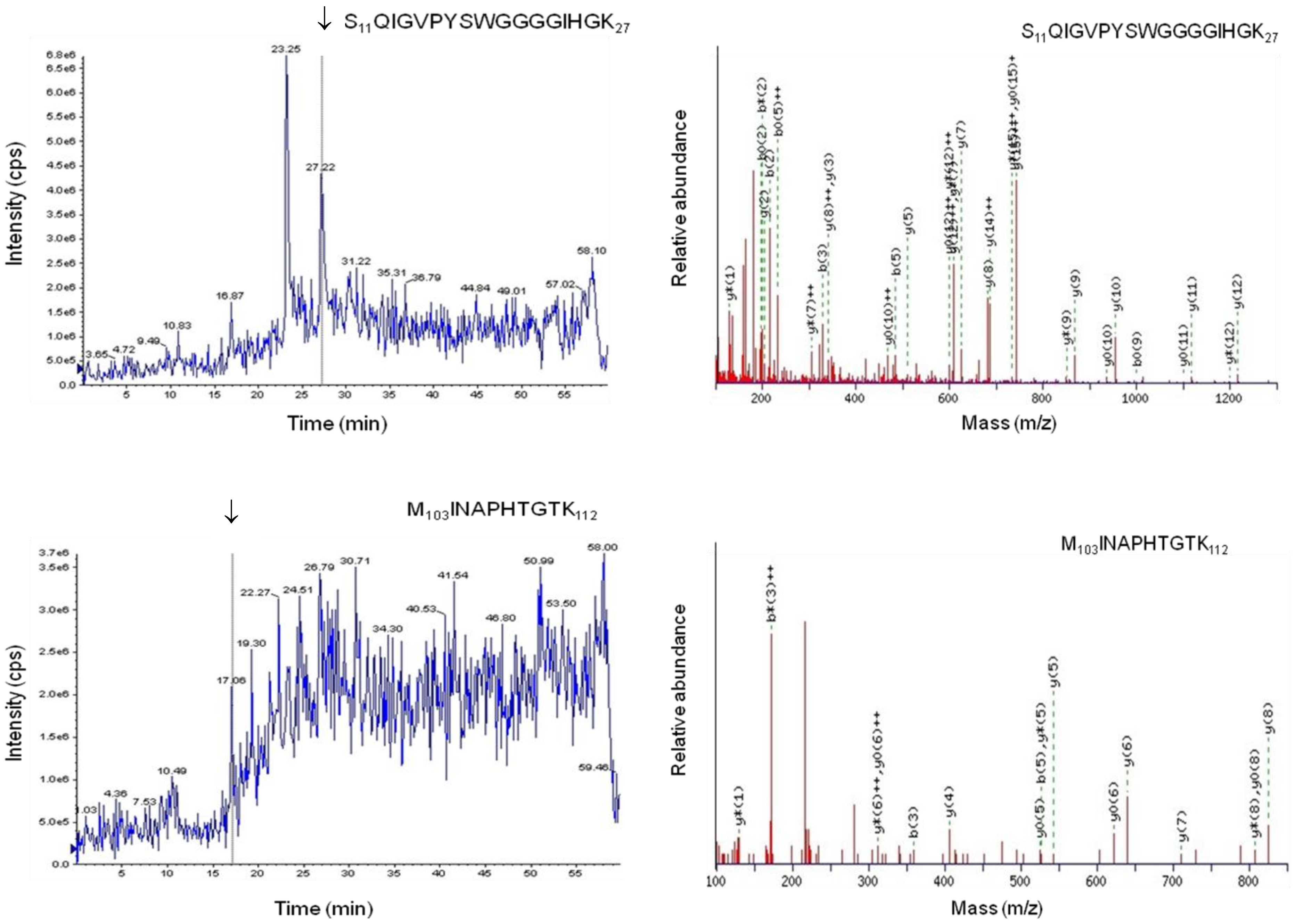
S11QIGVPYSWGGGGIHGK27
Intensity (cps)
Time (min)
S11QIGVPYSWGGGGIHGK27
Relative abundance
Mass (m/z)
M103INAPHTGTK112
Intensity (cps)
Time (min)
M103INAPHTGTK112
Relative abundance
Mass (m/z)

Fig. S4

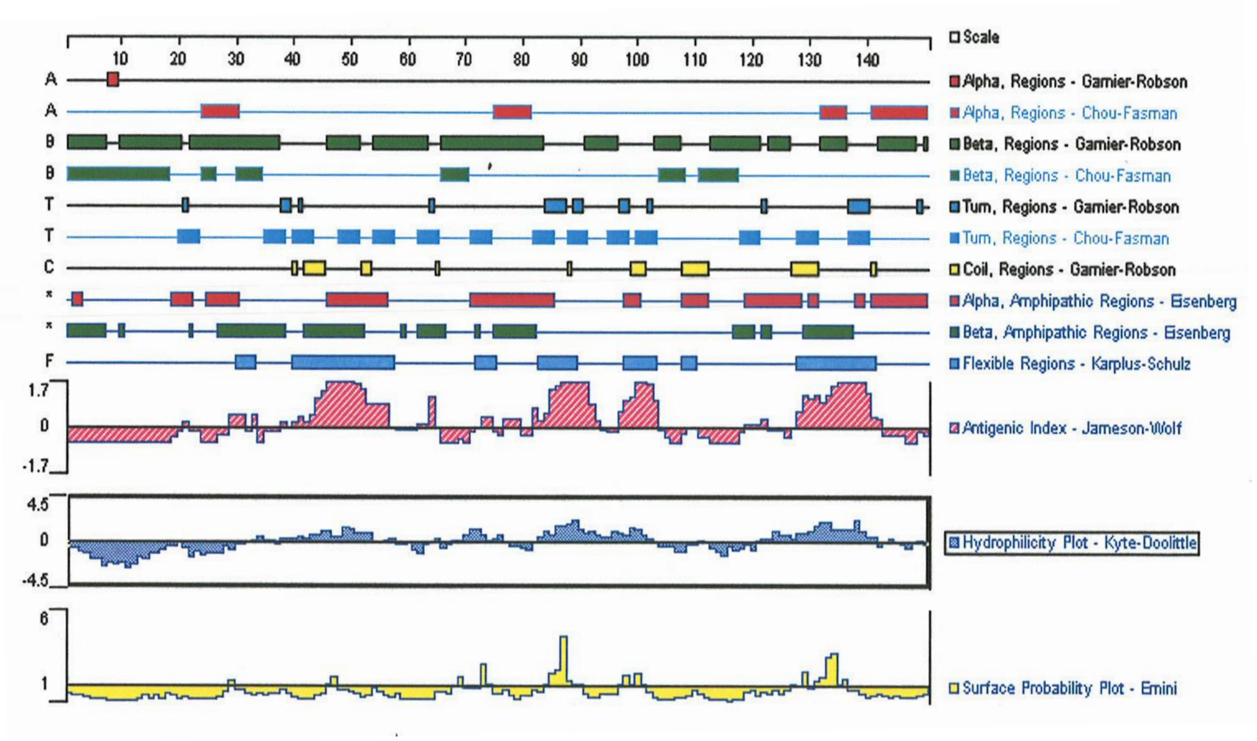

MKFFFTLALFCTLAISQVYCNGAAIVSAARSQIGVPYSWGGGGIHGKSRGIGEGANTVGFDCSGLAQYS
VYQGTHKVLARVASGQYSDPKCHHVAYGSHQPGDLVFFGNPIHHVGIVSAHGRMINAPHTGTKVREENI
GGDHIANVARCW

Fig. S5

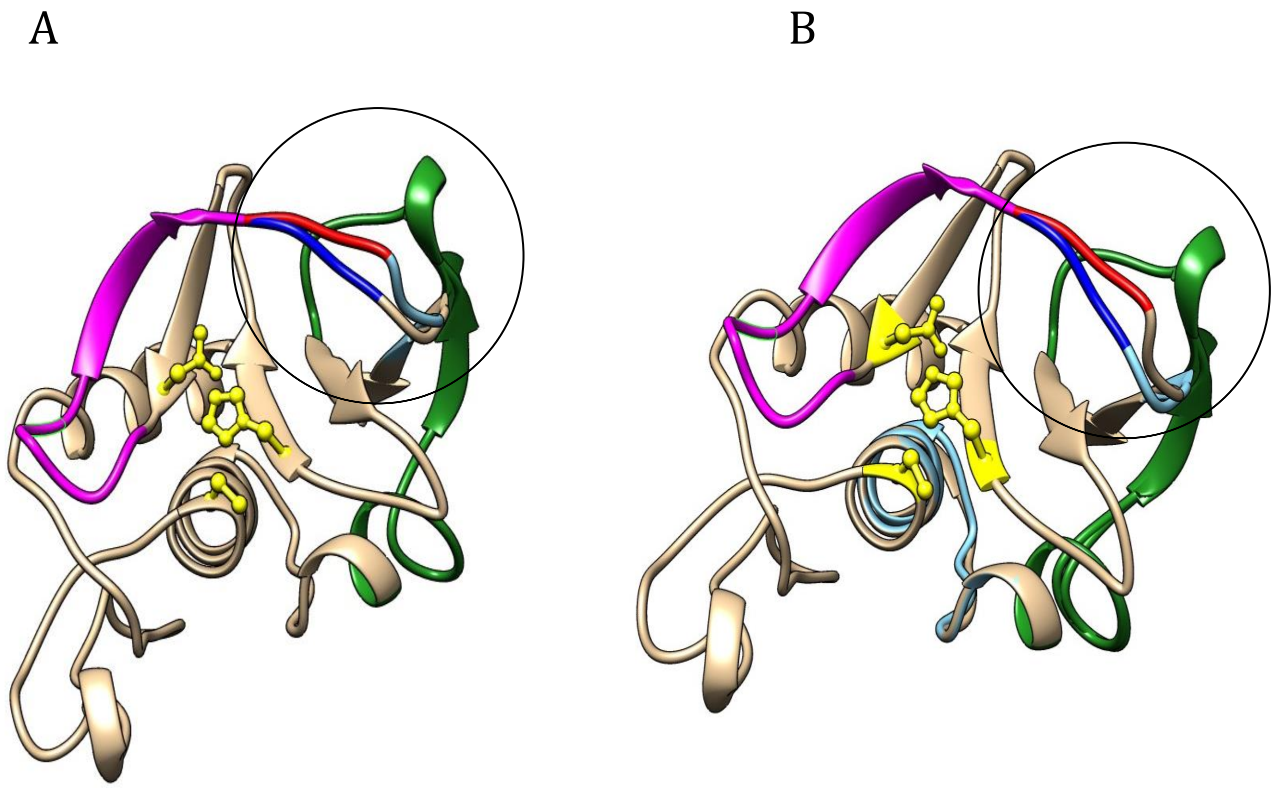

Fig. S6

A

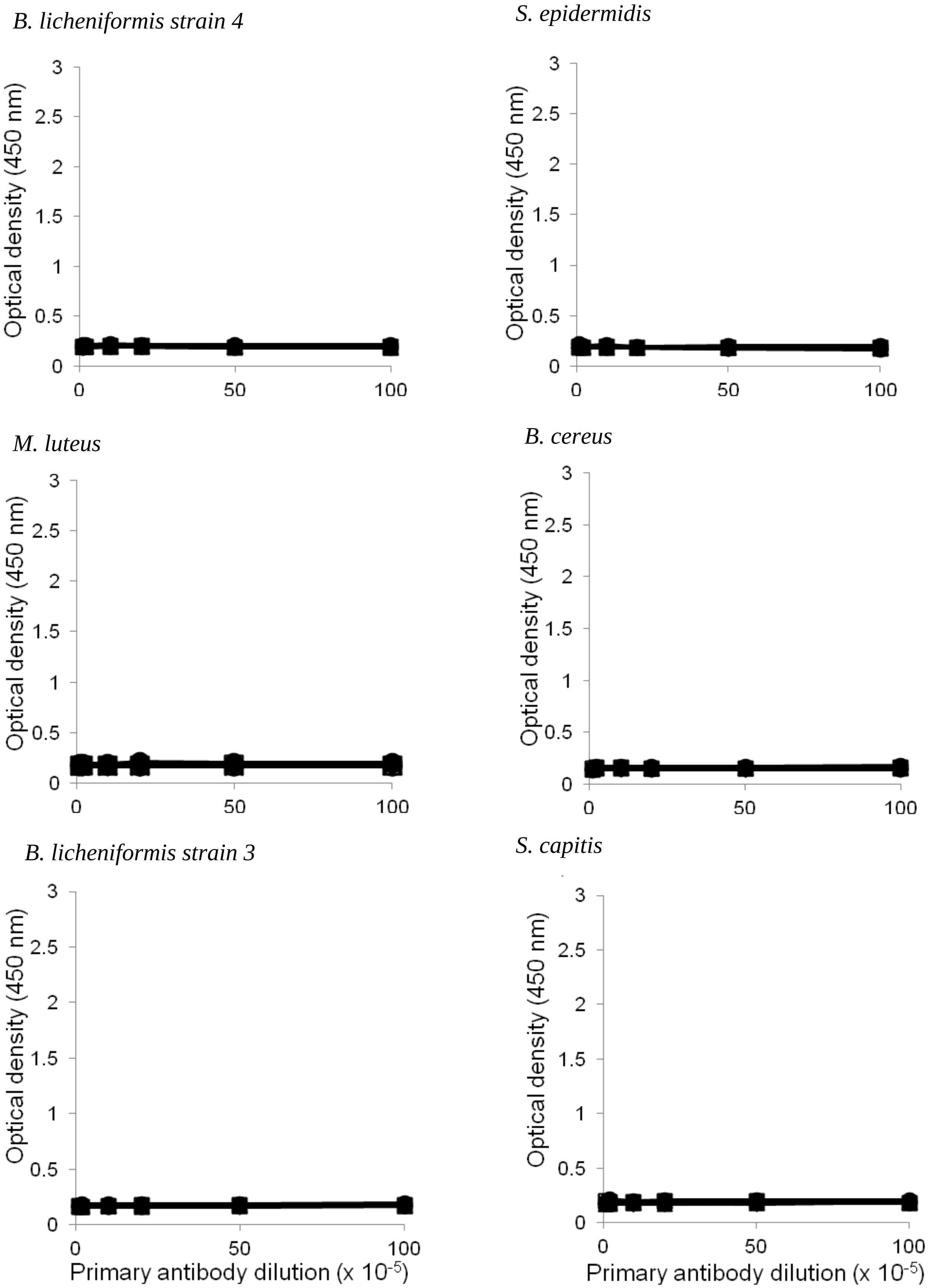
B. licheniformis strain 4
S. epidermidis
M. luteus
B. cereus
B. licheniformis strain 3
S. capitis
Optical density (450 nm)
0
0.5
1
1.5
2
2.5
3
0
50
100
Primary antibody dilution (x 10-5)

B

B. licheniformis strain 4

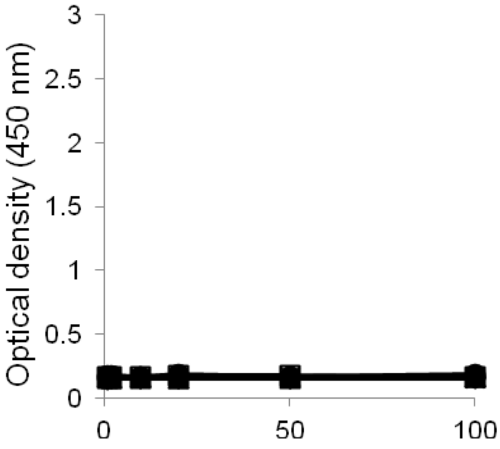

S. epidermidis

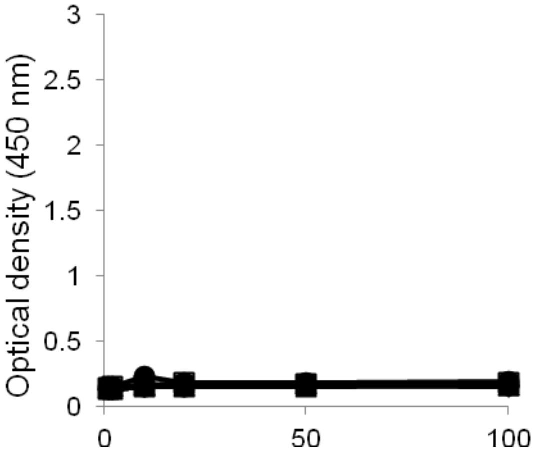

M. luteus

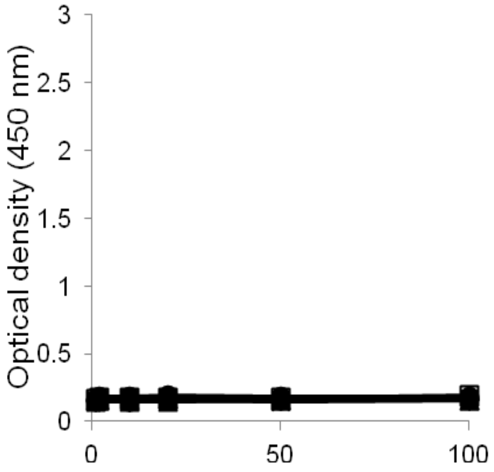

B. cereus

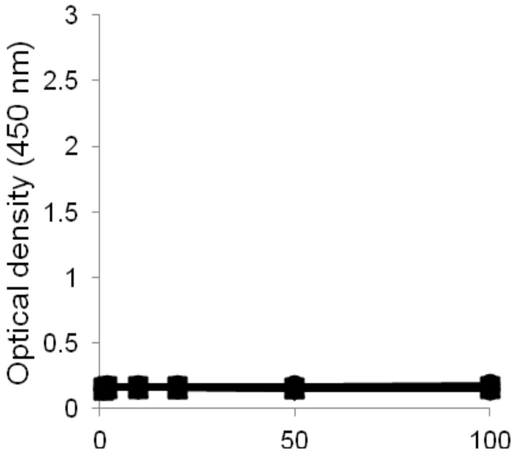

B. licheniformis strain 3

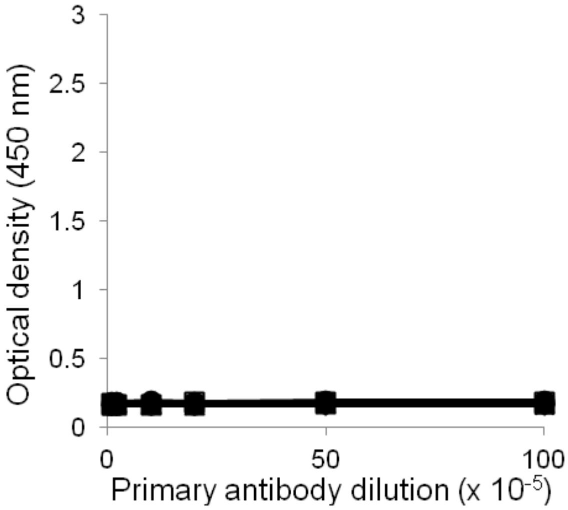

S. capitis

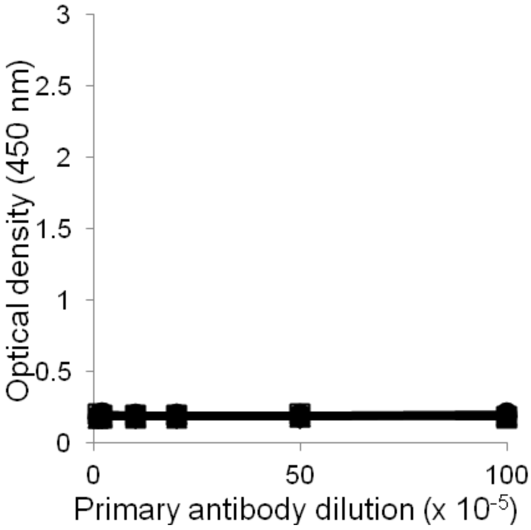

Fig. S7

```
                                                                                                          60
Rhagoletis zephyria RipB-like protein(XP017462582)   -GGSEIVAAARTQIGVPYSWGGGGWSGKSRGIQQGAHTVGFDCSGLAQYSVYHGTHKKIA
Rhagoletis zephyria RipB-like protein(XP017462583)   AGGAEIAAAARTQIGLPYSWAGGSWAGKSRGVLSGAHTVGFDCSGLAQYAVYQGTHKKIA
Blomia tropicalis LytFM homologue (CB282085)         AGGHEIVTAARSQLGVPYSWGGGNWAGKSKGIDSGAHTVGFDCSGLAQYAVYHGTHKKIA
Dermatophagoides pteronyssinus LytFM1 (AAN02509)     -NGAAIVSAARSQIGVPYSWGGGGIHGKSRGIGEGANTVGFDCSGLAQYSVYQGTHKVLA
Dermatophagoides pteronyssinus LytFM (AGV05390)      -NGAAIVSAARSQIGVPYSWGGGGIHGKSRGIGEGANTVGFDCSGLAQYSVYQGTHKVLA
Dermatophagoides farinae LytFM homologue (KN266412)  -DGSHIVKAARSQIGVPYSWGGGGIHGKSKGIGEGANIVGFDCSGLAQYSIYQGTHKTIA
Psoroptes ovis LytFM homologue (FR749374)            -NGAGIAAAARSQIGVPYSWGGGGIHGKSRGIGPGANIVGFDCSGLAQYSVYQGTHKVIA

                                                                                                         120
Rhagoletis zephyria RipB-like protein (XP017462582)  RVASAQYHDHQCHHVPYAQHQPGDLVFFAKGSDIHHVAIISGHNTMIEAPHTGAKVHETS
Rhagoletis zephyria RipB-like protein (XP017462583)  RVAGAQYADHQCHHVPYAQHQPGDLVFFNDGGSIHHVAVISGHDRMVHAPHTGDHVREAA
Blomia tropicalis LytFM homologue (CB282085)         RVASAQYADHQCHHVPYAQHLPGDLVFFNDGGSIHHVAIISGKNTMIHAPHTGDHVREAA
Dermatophagoides pteronyssinus LytFM1 (AAN02509)     RVASGQYSDPKCHHVAYGSHQPGDLVFF--GNPIHHVGIVSAHGRMINAPHTGTKVREEN
Dermatophagoides pteronyssinus LytFM (AGV05390)      RVASGQYSDPKCHHVAYGSHQPGDLVFF--GNPIHHVGIVSAHGRMINAPHTGTNVREEN
Dermatophagoides farinae LytFM homologue (KN266412)  RTAAAQYNDNHCHHVAYGSHQPGDLVFF--GNPIYHVGIVSAHGRMVNAPKPGTKVREEN
Psoroptes ovis LytFM homologue (FR749374)            RVAAAQYNDRQCHRVPFSQHQPGDVVFF--GNPPYHVGIVSAHNKMANAPKPGTTVREES

                                                                134
Rhagoletis zephyria RipB-like protein (XP017462582)  VRAAERMANVARCF
Rhagoletis zephyria RipB-like protein (XP017462583)  VYVKGRMDLVTR--
Blomia tropicalis LytFM homologue (CB282085)         VYVKGRMSTVQRCF
Dermatophagoides pteronyssinus LytFM1 (AAN02509)     IG-GDHIANVARCW
Dermatophagoides pteronyssinus LytFM (AGV05390)      IW-SDHIANVARCW
Dermatophagoides farinae LytFM homologue (KN266412)  IW-SYHISHVARCW
Psoroptes ovis LytFM homologue (FR749374)            IW-GYHLGNVARCW
```

Table S1: Summary of proteins other than LytFM1 present in the bacterial culture supernatants of the HDM-associated *B. licheniformis strain 1*, *B. licheniformis strain 2* and *S. aureus* following analysis by MS.

Microorganism (source of secreted proteins)	Protein identity and accession number	Size of protein	Microorganism possessing protein	Peptide sequence	Probability-based score*	Rank
B. licheniformis 1	Trypsin-like serine protease (Q7Z163)	29 kDa	*Dermatophagoides pteronyssinus*	YPTIYSNVANLR	0.00100	1
B. licheniformis 1	17 kDa surface antigen (NC014117.1)	17 kDa	*Burkholderia spp.*	MGTVDSVR	0.05500	1
B. licheniformis 1	Spore coat protein E (A0RHE2)	20 kDa	*Bacillus thuringiensis*	VNYTDEVSIGYR	0.00095	1
B. licheniformis 2	Der p 1 allergen (Q3HWZ5)	34 kDa	*Dermatophagoides pteronyssinus*	QIEYIQHNGVVQESYYR	0.00024	1
B. licheniformis 2	Der p 2 allergen (B0FRE2)	15 kDa	*Dermatophagoides pteronyssinus*	SENVVVTVK	0.05600	1
S. aureus	Der p 1 allergen (Q3HWZ5)	34 kDa	*Dermatophagoides pteronyssinus*	GIEYIQHNGVVQESYYR	0.016	1
S. aureus	Putative uncharacterised protein (Q72X58)	16 kDa	*Bacillus cereus*	GSSLGTQSYTGIIEAAGR<br>QATVVMTYER	5.1e-005<br>0.00027	1

*****Denotes the score used to determine if a positive hit is above the significance threshold which is generally $p < 0.05$, where p is the probability that the positive hit occurred by chance. The rank "1" was assigned to a peptide that gave rise to one of the fifteen most intense peaks based on calculations performed with Mascot.
